# Prevalence Soil Transmitted Helminthiasis and malaria co-infection among pregnant women and risk factors in Gilgel Gibe dam Area, Southwest Ethiopia

**DOI:** 10.1186/1756-0500-6-263

**Published:** 2013-07-09

**Authors:** Million Getachew, Ketema Tafess, Ahmed Zeynudin, Delenesaw Yewhalaw

**Affiliations:** 1 Department of Biomedical Science, School of Health and Hospital, Adama Science and Technology University, Asella, Ethiopia; 2 Department of Medical Laboratory Sciences and Pathology, College of Public Health and Medical Sciences, Jimma University, Jimma, Ethiopia; 3 Department of Biology, College of Natural Sciences, Jimma University, Jimma, Ethiopia

**Keywords:** Co-infection, Malaria, Pregnant women, Soil transmitted helminths, Ethiopia

## Abstract

**Background:**

Malaria and Soil Transmitted Helminthiasis (STH) are co-endemic and major public health problems in Ethiopia. The aim of the study was to assess the prevalence of malaria and STHs co-infection and to determine the association risk factors.

**Methods:**

A cross-sectional community based study was conducted on 388 pregnant women living in three districts around Gilgel Gibe Dam area, southwestern Ethiopia. Socio-demographic and socio-economic data, single stool sample and blood sample were collected from each participant.

**Results:**

The prevalence of STH and malaria was 159 (41%) and 45 (11.6%), respectively and the prevalence of STHs/malaria co-infection was 30 (7.7%). Hookworm was the most prevalent 114 (29.4%) soil transmitted helminthiasis infection followed by *Ascaris lumbricoides (A. lumbricoides)* 58 (15%) and *Trichuris trichiura* (*T. trichiura)* 13 (3.4%). Habit of eating soil (Adjusted Odds Ratio (AOR) = 4.64, 95% CI: 1.50-14.36, P=0.008), presence of stagnant water near study participants’ house (AOR=2.99, 95% CI: 1.28-6.99, P=0.012) and habit of using human feces as a fertilizer (AOR= 5.34, 95% CI: 1.99-14.28, P<0.001) were found to be significantly associated with malaria and STH co-infection among the pregnant women. Hookworm parasitic load was positively correlated with malaria parasitic load (r = 0.299, P<0.001) while *A. lumbricoides* parasitic load was negatively correlated with malaria parasitic load (r = −0.095, P<0.001).

**Conclusion:**

Intestinal parasite and/or malaria co-infection is a health problem among pregnant women living around Gilgel Gibe dam area. Therefore, intervention including improving sanitation, removing stagnant water, and health education to the pregnant women should be given.

## Background

Parasitic diseases pose major obstacles to health growth and socio-economic development in developing countries. Parasitic diseases such as malaria are life threatening as well as the leading cause of mortality in endemic countries; more severe to some risky groups, like pregnant women [1]. In pregnancy, there is a transient depression of cell-mediated immunity that allows fetal allograft retention which in the other hand interferes with resistance to various infectious diseases such as malaria [2,3]. On the top of host, pregnant women, immunossupression; studies showed that immunological interactions between protozoan and helminths infection can intensify the impact of parasitic infection when they co-exist [4,5].

Immunological factors are expected to influence rates of co-infection because helminths modulate host immune responses both to themselves, and to concurrent infections. That is why pregnant women, immunologically compromised, are highly susceptible for parasitic infections such as malaria and Soil transmitted helminthiasis [6]. Actually there are still controversies concerning the biological association between helminthes and protozoa parasites [7]; WHO, [8]; Murray, [9]; Nacher et al., [10].

It was estimated that over a third of the world's population, mainly in the tropics and sub-tropics, is infected with parasitic helminthes and Plasmodium species [11] and an estimated 40 million pregnant women were infected with STHs and schistosomes globally [12]. Hospital based studies conducted among Nigerian pregnant women indicated more than 18% of co-infection [13]. In a similar study done in Ghana, prevalence of co-infection was found to be 16.6% and women with intestinal helminth infection(s) were 4.8 times more likely to have malaria infection (Yatich et al., [14]).

In Ethiopia, the study done in Alaba Kulito Health Center, southern Ethiopia, reviled that only few cases of helminth and malaria co-infected patients showed signs and symptoms of severe malaria as compared with severe manifestation in patients infected with plasmodium parasite alone (Tadege et al., [15]). This indicates that helminth infection provides some degree of protection against the development of severe malaria which is also in agreement with the previous reports (Nacher et al., [10]).

Generally, there is poor understanding of the association between malaria and helminths infection and the basic factors for their co-occurrence especially in pregnant women. There is no satisfactory published literature on the prevalence and associated risk factors for co-infection of malaria and intestinal helminths among pregnant women in Ethiopia in general and in Gilgel Gibe dam area in particular. Therefore, this study was intended to investigate the prevalence of malaria and soil transmitted helminthes co-infection among pregnant women in Gilgel Gibe dam area and to assess the possible risk factors for these co-infections.

## Methods

### Study design

A cross-sectional community based study was conducted from August to September, 2011 in Gilge Gibe Dam area, 260 km south-west of Addis Ababa, near Gilgel-Gibe hydroelectric dam which started operating in 2004. The total population was 55,000. The study area lies between latitudes 7°42'50"N and 07°53'50"N and between longitudes 37°11'22"E and 37°20'36"E, at an altitude of 1,672-1,864 m above sea level. The area has a sub-humid, warm to hot climate, receives between 1,300 and 1,800 mm of rain annually and has a mean annual temperature of 19°C. The rainfall pattern of the area is similar to other parts of Ethiopia with the long rainy season starting in June and extending up to September, while the short rainy season begins in March and extends to April/May. Participants from six rural and six urban kebeles were assessed through house to house visit. The study participants were then randomly selected using systematic random sampling from the sampling frame which was constructed after identifying the pregnant women.

### Data collection and processing

#### Laboratory work

After getting their written consent, stool samples were collected from participants. The study participants were provided with labeled screw caped stool cap and informed on how to collect about 5 gram stool sample. The collected stool samples were soon transported to Jimma university clinical laboratory where it was processed following the standard procedure of McMaster concentration technique. The type of parasite and parasitic load were recorded in the prepared laboratory format.

Within the same day when the participants donate their stool sample, capillary blood sample was also collected from all the 388 pregnant women following aseptic technique. Then, the finger was cleansed with alcohol-moistened swab, dried with a piece of dry cotton, punctured with a disposable blood lancet. Through wiping off the first drop of blood, thick and thin films were made on the same slide. After being air-dried in a horizontal position, the slides were placed in a slide box and carefully transported to Asendabo Health Center where parasitological test was carried by experienced laboratory technicians and technologists.

Before staining the blood films, the thin blood films were fixed in methanol for 30 sec. Then smears were stained with 10% Giemsa solution for 10 minutes. The staining techniques and blood film examination was conducted according to WHO guideline. Microscopic examination of thick films using high power magnification for the presence of parasites and parasite species identification using thin films under 100x oil immersion objective were done by experienced laboratory technicians and technologists. In addition to the qualitative examination, parasitic load was determined following WHO guide line.

### Questionnaire survey

A semi structured questionnaire was developed and administered to identify possible risk factors of malaria/STH co-infection in the study area. The questionnaires address the individuals’ socio-demographic information, use of malaria and STH preventive measures, housing conditions, their knowledge and attitude concerning malaria and soil transmitted helminthes transmission and other related issues to assess some possible risk factors for the co-infection.

### Data analysis

Data from both the laboratory and survey were checked and cleaned for completeness and consistency. Data were then analyzed using SPSS version 16.0 software package. For the analysis of demographic data descriptive statistics was employed. Point estimation of prevalence and intensity of STH calculated based on the stool and blood sample results. The comparison of prevalence of STH and malaria co-infection between different explanatory variables was done using chi-squire (*X*^2^) test Odds ratios (OR) with a 95% confidence interval were computed to compare the strength of association between explanatory and outcome variable. Multivariable logistic regressions were also employed for those variables that had significant association with disease outcome to determine the main predictors of infection. P-value ≤ 0.05 was considered significant during the analysis.

### Ethical consideration

Ethical clearance of the study was obtained from the Research Ethics Review Board of Jimma University. Permission from the community was sought before initiating the study by communicating the responsible zonal and district administrative offices through official letters from Jimma University. Similarly, community agreement and local oral consent was sought from village leaders through meetings with villagers. Individual informed oral and written consent were sought from each pregnant woman in the local language, *Afaan Oromo*, for all literate pregnant women. An independent literate witness from village leaders confirmed verbal consent for illiterate pregnant woman after the objectives and the nature of the study were explained to the participants so as to get their oral and written consent to be involved in the study voluntarily. Data collected during the survey from each study participant and results of laboratory tests were kept confidential. Results of participants with parasitic infections, malaria and/or Malaria- STH infection were sent, as soon as possible, to nearby health facilities for treatment and medical consultation in the ANC. Those pregnant women found infected were referred for treatment.

## Results

### Socio demographic and socio economic characteristics of respondents

Non response rate was of 1.3%. From the total of 388 pregnant women, who responded, 195 (50.3%) live in rural areas and the rest 193 (49.7%) live in urban areas. The minimum and maximum ages were 16 and 40, respectively with mean age of 25 years. During the study 88 (22.7%), 157(40.5%), 143 (36.8%) were in their first, second and third trimester, respectively. The proportion of primigravida (pregnant for the first time) and multigravida were 125(32.2%) and 263(67.8%), respectively. Majority of the participants 372 (95.9%) were Oromo in ethnicity and more than 92% of the respondents were Muslims (Table 1).

**Table 1:** Socio demographic characteristics of respondents in Gilgel Gibe hydropower dam area, Southwest Ethiopia, August to September 2011

**Residence**	**Number**	**Percent**
Urban	193	49.7
Rural	195	50.3
**Age groups**		
16-20	128	33
21-25	115	29.6
26-30	110	28.3
31-35	27	7
36-40	8	2.1
**Ethnicity**		
Oromo	372	95.9
Amhara	4	1.1
Yam	7	1.8
Gurage	4	1.1
kembata	1	0.2
**Religion**		
Muslim	359	92.5
Orthodox	21	5.4
Protestant	6	1.6
Catholic	2	0.5
**Parity**		
Primigravid	125	32.2
Multigravid	263	67.8
**Occupation**		
Housewife	191	49.2
Farmer	176	45.3
Daily laborer	4	1.1
Civil servant	13	3.31
Business man	4	1.1
**Educational status**		
Illiterate	263	67.8
Read and write only	5	1.3
Primary school	83	21.4
Secondary school	18	4.6
Above Secondary school	19	4.9
**Trimester**		
First	88	22.7
Second	157	40.5
Third	143	36.8

### Prevalence of malaria and STH co-infection and associated risk factors among pregnant women

**Figure 1:**
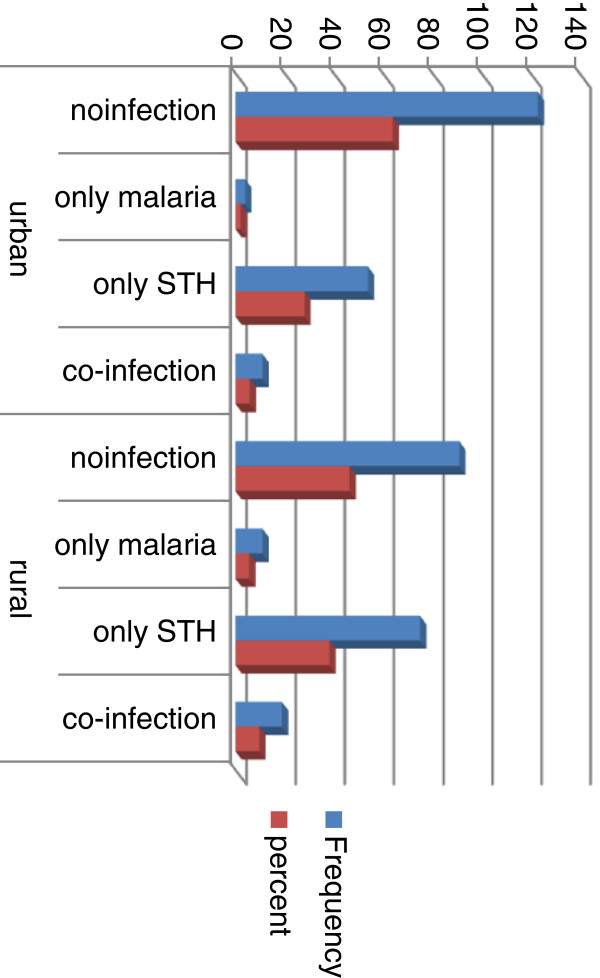
Proportion of malaria, STH infection and co-infection among pregnant women in Gilgel Gibe hydropower dam area, Southwest Ethiopia, August to September 2011.

Overall, STH and malaria prevalence among pregnant women in the study area were 159 (41%) and 45 (11.6%), respectively. Parasitic infection was detected in 65 (33.9%) and 97 (48%) urban and rural residents pregnant women, respectively. STHs detected in the stools of the pregnant women were *Hookworm 114* (29.4%), *A.lumbricoides 58* (14.9%), *T.trichuria 13* (3.4%), *H.nana 8* (2.1%), *E.vermicularies 5*(1.3%). The prevalence of STH and malaria co- infection among pregnant women was 30 (7.7%) (Table 2 and Figure 1).

**Table 2:** Prevalence of mono, dual and triple intestinal parasitic infection in pregnant women in Gilgel Gibe hydropower dam area, Southwest Ethiopia, August to September 2011

**Character (variable)**	**Number of individual infected**	
	**Urban**	**Rural**
**Mono infection**		
Hookworm	38(44.7)	47(55.3)
*A.lumbricoides*	14(45.2)	17(54.8)
*T.trichiuria*	0	1(100)
*H.nana*	0	6(100)
*E. vermicularis*	0	1(100)
**Dual infection**		
Hookworm *and A. lumbricoides*	7(36.9)	12(63.2)
Hookworm *and T.trichiuria*	0	2(100)
Hookworm *and E. vermicularis*	1(33.3)	2(66.7)
*A.lumbricoides and T.trichiuria*	0	3(100)
*T.trichiuria and E. vermicularies*	0	2(100)
*H.nana and E.vermicularies*	1(33.3)	2(66.7)
**Triple infection**		
Hook worm*, A.lumbricoides, E. vermicularies*	1(100)	0
Hookworm*, A.lumbricoides, T.trihiuria*	2(50)	2(50)

Socio demographic and others variables were analyzed in relation to Malaria and STH co-infection by bivariate and multivariate logistic regression analyses model. In the bivariate logistic regression analysis, Malaria and STH co-infection was associated significantly with thatched roof, presence of stagnant water in the residence area, use of human faeces as fertilizer, and habit of soil eating (Table 3).

**Table 3:** Association between different risk factors and malaria/STH co-infection among pregnant women in Gilgel Gibe hydropower dam area, Southwest Ethiopia, Chi square analysis, August to September 2011

**Variables**	**Total**	**Malaria and STH co-infection**	**P – value**
**Residence**			
Urban	193	11	0.101
Rural	195	19	
**House type**			
Thatched	148	16	0.058
Corrugated iron sheet	240	14	
**Presence of stagnant water**			
Yes	62	10	0.011
No	326	20	
**Age group**			
16-20	128	8	0.826
21-25	115	10	
26-30	110	10	
31-35	27	2	
36-40	8	0	
**Utilization of human feces as fertilizer**			
Yes	32	7	0.007
No	356	23	
**Habit of eating soil**			
Yes	22	5	0.020
No	366	25	
**Trimester**			
First trimester	88	7	0.675
Second trimester	157	10	
Third trimester	143	13	
**Parity**			
Primigravide	125	8	0.324
Multigravide	263	22	

Habit of eating soil (AOR = 4.64, 95% CI: 1.50-14.36, P=0.008), Presence of stagnant water near study participants’ house (AOR=2.99, 95% CI: 1.28-6.99, P=0.012) and habit of using human feces as a fertilizer (AOR= 5.34, 95% CI: 1.99-14.28, P<0.001) were found to be significantly associated with malaria and STH co-infection among the pregnant women on multivariate logestic regression model (Table 4).

**Table 4:** Parameter estimates from univariate and multivariable logistic regression model predicting the likelihood that a pregnant woman is co-infected with STH and malrai, Gilgel Gibe Dam area, Southwest Ethiopia, August to September 2011

**Variables**	**Malaria and STH co-infection**		**COR**	**CI**	**AOR**	**CI**	**p-value**
	**Total**	**Co-infected**					
**Residence**							
Rural	195	19	1.77	0.82-3.81			
Urban	193	11	1				
**House type**							
Thatched	148	16	1.96	0.92-4.13			
Corrugated iron sheet	240	14	1				
**Presence of stagnant water**							
Yes	62	10	2.94	1.30-6.64	2.89	1.28-6.99	0.012
No	326	20	1			1	
**Utilization of human feces as fertilizer**							
Yes	32	7	4.05	1.59-10.36	5.34	1.99-14.28	0.001
No	356	23	1			1	
**Habit of eating soil**							
Yes	22	5	4.01	1.37-11.77	4.64	1.50-14.36	0.008
No	366	25	1			1	
**Trimester**							
Third trimester	143	13	1.16	0.29-2.15			
Second trimester	157	10	0.78	0.44-3.02			
First trimester	88	7	1				
**Parity**							
Multigravide	263	22	1.33	0.58-3.09			
Primigravide	125	8	1				

*COR* Crude odds ratio*, AOR* Adjusted Odds Ratio*, C.I* Confidence Interval*.*

Pregnant women infected with STH were likely to be three times more infected with malaria compared to these pregnant women with no STH infection (OR=3.3, 95% CI: 1.72-6.40, P<0.001). Infections with hook worm (OR=1.540, 95% CI: 0.81-2.94), *A.lumbriciodes* (OR= 3.95, 95% CI: 1.98-7.88) and *T.trichuira* (OR=21.19, 95% CI: 6.21-72.26) were found to increase malaria prevalence. There was statistically significant association between number of helminthes parasite species and malaria infection rate (P<0.001) and it was found that the rate of malaria infection was higher in those pregnant women infected with more than one helminth species (Table 5). At an individual parasite load level, Hookworm parasitic load was positively co-related to malaria parasitic load (r = 0.299, P<0.001), in contrary *A. lumbricoids* parasitic load was negatively co-related to malaria parasitic load (r= −0.095, P<0.001).

**Table 5:** Association between number of helminthes species and malaria infection rate among pregnant women in Gilgel Gibe hydropower dam area, Southwest Ethiopia, August to September 2011

**Parasitic infection**			**Total**	**Chi-square test Value**	**P**
**STH species**	**Malaria**				
	**Negative**	**Positive**			
Single	117	10(7.9%)	127	4.79	<0.001
Double	25	2(7.4%)	27		
Triple	4	1(20%)	5		

## Discussion

Most of the time malaria and STH infections share endemicity in Ethiopia. The prevalence of malaria/STH co- infection among the pregnant women in our study was 7.7% with rate of co-infection higher in rural (9.7%) than urban (5.7%) pregnant women. Though it is not statistically significant, co-infection was more prevalent in those within the age group range of 26–30 year which was different from the previous report from Nigeria where pregnant women less than 20 were the most co-infected group [13]. The prevalence of co-infection in our study was lower than the prevalence of co-infection reported from Thialand [16], Ghana (Yatich et al., [14] and Nigeria [13]).

There was strong association between malaria and STH infection in which those pregnant women infected with STH were 3.3 times likely to be infected with malaria which is almost similar to a study from Ghana where women with intestinal helminth infection (s) were 4.8 times more likely to have malaria infection (Yatich et al., [14]. In this study hookworm parasitic load was positively co-related to malaria parasitic load and this result was comparable with the previous studies which suggested that STH infections may increase the risk of malaria infection [7,17] and especially hookworm aggravates the severity of malaria [18,19]. In contrary *A. lumbricoids* parasitic load was negatively co-related to malaria parasitic load; therefore as the parasitic load of *A. lumbricoids* increases the severity and parasitic load of malaria decreases. This finding was comparable with the studies which showed the protective effect of *A. lumbricoids* to reduce the severity of malaria [16,18].

In the current study, the overall STH prevalence, 159 (41%), was lower than the findings from Bushulo health center, Southern Ethiopia 58.2% [15] and Thailand 70% [16] but was higher than the findings reported from Kenya 25.7% [20] and Ghana 36.3% (Yatich et al., [14]. Hookworm was found to be the most prevalent STH. The prevalence of Hookworm, *A.lumbricoieds* and *T.trichuiria* were 29.4%, 15% and 3.4%, respectively. This result was different from the prevalence of national report [21] as well as study done in Nigeria [13]. This difference may be due to the difference in the subjects of the studies in which in the national reports all community members were used; and different community members are differently exposed to parasitic infection and thus the prevalence differed. In addition to that the difference in soil type among these three studies may have changed the prevalence of infection. Prevalence of hook worm was similar to prevalence the report from Butajira, Ethiopia [21] and Kenya [19] but lower than the prevalence reported from Thailand 42.8% [15] and Peru 47.22% [22].

Malaria is the most serious public health problem in Ethiopia where it is highly distributed throughout the country. In our study malaria prevalence in Gilgel-Gibe dam area was 11.6% with *P.falciparum* and *P.vivax* proportion of 71.1% and 15.6%, respectively; whereas mixed infection with both species and maximum parasitic load were 13.3% and 36,480 parasites/μL, respectively. Malaria prevalence found in this study was by far lower than the results of similar studies done in Nigeria [13] and Burkina Faso [23], but almost similar to malaria prevalence reported from Sudan [24]. This difference may be because of climatic and life style difference of the communities. The malaria parasite species distribution was different from the previous study done in the same area with different study subjects, where *P.vivax* was reported to be more prevalent than *P.falciparum*[25].

In this study presence of stagnant water, habit of eating soil and habit of using human feces as a fertilizer were associated risk factors for malaria and STH co-infection among pregnant women in Gilgel Gibe dam area in which pregnant woman who lives in house which is near stagnant water was three times more likely to be co-infected. Study done in Thailand, Ghana and Nigeria however identified parity, low income, being young age and marital status (being single) as possible factors associated with malaria STH co-infection [13]; Yatich et al., [14]. Therefore, targeting the possible risk factors identified in this study may reduce malaria and STH co-infection in pregnant women.

## Conclusion

Intestinal parasite and/or malaria co-infection is a health problem among pregnant women living around Gilgel Gibe dam area. Therefore, intervention including improving sanitation, removing stagnant water, and health education to the pregnant women should be given.

## Competing interest

The authors declare that they have no competing interests.

## Authors’ contributions

MG conceived the study, designed, participated in data collection, conducted data analysis, drafted and finalized the manuscript for publication. DY, AZ and KT assisted in data collection and reviewed the initial and final drafts of the manuscript. MG, DY, AZ and KT interpreted the results, and reviewed the initial and final drafts of the manuscript. All authors read and approved the final manuscript.
